# Identification of Eight High Yielding Strains via Morpho-Molecular Characterization of Thirty-Three Wild Strains of *Calocybe indica*

**DOI:** 10.3390/foods12112119

**Published:** 2023-05-24

**Authors:** Manoj Nath, Anupam Barh, Annu Sharma, Parul Verma, Rakesh Kumar Bairwa, Shwet Kamal, Ved Prakash Sharma, Sudheer Kumar Annepu, Kanika Sharma, Deepesh Bhatt, Pankaj Bhatt, Dharmesh Gupta, Akoijam Ratankumar Singh

**Affiliations:** 1 ICAR-Directorate of Mushroom Research, Solan 173 213, India; 2 ICAR-Indian Institute of Soil and Water Conservation, Dehradun 248 195, India; 3 Department of Plant Pathology, College of Horticulture, Dr. Yashwant Singh Parmar University of Horticulture and Forestry, Solan 173 230, India; 4 ICAR-Indian Institute of Soil and Water Conservation, Research Center, Ooty 643 006, India; 5 Department of Biotechnology, Shree Ramkrishna Institute of Computer Education and Applied Sciences, Faculty of Science, Sarvajanik University, Surat 395 001, India; 6 Department of Agricultural & Biological Engineering, Purdue University, West Lafayette, IN 47906, USA; 7 ICAR Research Complex for North Eastern Hill Region, Manipur Centre Lamphelpat, Imphal 795 004, India

**Keywords:** tropical mushroom, genetic improvement, high yielding strains, SRAP marker, genetic diversity, bioactive compounds

## Abstract

*Calocybe indica*, generally referred as milky mushroom, is one of the edible mushroom species suitable for cultivation in the tropical and sub-tropical regions of the world. However, lack of potential high yielding strains has limited its wider adaptability. To overcome this limitation, in this study, the germplasms of *C. indica* from different geographical regions of India were characterized based on their morphological, molecular and agronomical attributes. Internal transcribed spacers (ITS1 and ITS4)-based PCR amplification, sequencing and nucleotide analysis confirmed the identity of all the studied strains as *C. indica*. Further, evaluation of these strains for morphological and yield parameters led to the identification of eight high yielding strains in comparison to the control (DMRO-302). Moreover, genetic diversity analysis of these thirty-three strains was performed using ten sequence-related amplified polymorphism (SRAP) markers/combinations. The Unweighted Pair-group Method with Arithmetic Averages (UPGMA)-based phylogenetic analysis categorized the thirty-three strains along with the control into three clusters. Cluster I possesses the maximum number of strains. Among the high yielding strains, high antioxidant activity and phenol content was recorded in DMRO-54, while maximum protein content was observed in DMRO-202 and DMRO-299 as compared with the control strain. The outcome of this study will help the mushroom breeders and growers in commercializing *C. indica.*

## 1. Introduction

*Calocybe indica* (P&C), commonly known as milky mushroom, is a tropical edible mushroom, originating in the Indian sub-continent. Unlike the common white button mushroom, *C. indica* requires warm and humid tropical climate for cultivation. With robust yield, attractive color, culinary delicacy and nutritional properties, milky mushroom has attracted the prospective mushroom growers, especially in the southern part of India [1,2,3]. It is one of the five major commercial mushrooms grown in India and comprises about 3 per cent of total production [4]. Longer shelf-life and white color of the fruit body are the key attractive features of *C. indica* which increase its demand among mushroom growers and consumers [5,6]. Moreover, the morphological resemblance of *C. indica* with *Agaricus bisporous* (common white button mushroom) augmented its popularity in India [3]. *C. indica* also possesses various medicinal and therapeutic applications. A detailed review on the account of nutritional and medicinal properties of *C. indica* was presented by Shashikant et al. [7] in which its potential anti-oxidant, anti-cancer, anti-obesity, hepatoprotective and anti-aging activities were reported. Despite its importance, very few strains were reported for commercial cultivation in India *viz.* APK-2 [8,9], Directorate of Mushroom Research (DMR)-Milky-985, DMR-Milky-334 (DMRO-302) [10,11]. In addition to this, high yielding strains were also reported by different researchers from different geographical regions of India *viz*. Jharkhand [12], Uttar Pradesh [13], Tamil Nadu [14], and Punjab [15].

A review of the literature indicates that most research on *C. indica* focused on cultivation and evaluation trials with limited number of strains at a particular geographical region. Thus, no systematic efforts were undertaken to study the genetic diversity in the *C. indica* gene pool. To initiate a systematic genetic improvement program, it is imperative to understand the existing genetic diversity in the targeted mushroom species. The potential of *C. indica* to grow at higher temperatures highlights its importance in expanding the mushroom industry in the tropical regions of India. In order to develop high yielding strains for the Indian context, strains from different geographical regions have been collected and assessed for their genetic diversity. With the advancement of molecular biology, DNA-based markers from genic regions have now been increasingly integrated with the analysis of genetic diversity in breeding programs. Among the series of molecular markers, SRAP (Sequence Related Amplified Polymorphism) is a recent marker that basically analyzes polymorphism in the exonic region of the genome. SRAP markers are considered efficient, effective, robust, inexpensive and reproducible marker technology [16]. These markers amplify the open reading frame (ORF) along with the intron and promoter region of the DNA [17]. This marker system is well established in analyzing the genetic diversity and DNA fingerprinting of different mushroom strains *viz*. *Lentinula edodes* [18,19], *Auricularia* spp. [20,21,22], *Lepista nuda* [23] and *Pleurotus* spp. [24,25,26]. Recently, SRAP markers/combinations were used in the genetic diversity analysis of twenty-four different strains of *Cyclocybe chaxingu* [27].

In the present study, we have performed an internal transcribed spacer (ITS)-based molecular identification of thirty-three wild strains of *C. indica* and evaluated their morphological and agronomic parameters through cultivation trial. Further, these strains were subjected to genetic diversity analysis using ten SRAP combinations/markers. In addition, eight high yielding strains identified in the cultivation trial were subjected to proximate analysis to understand their edibility and nutritional properties in comparison to the control strain (DMRO-302). The experimental results will accelerate *C. indica* breeding programs in India and also help to conserve the genetic resources.

## 2. Materials and Methods

### 2.1. Strains

**Figure 1:**
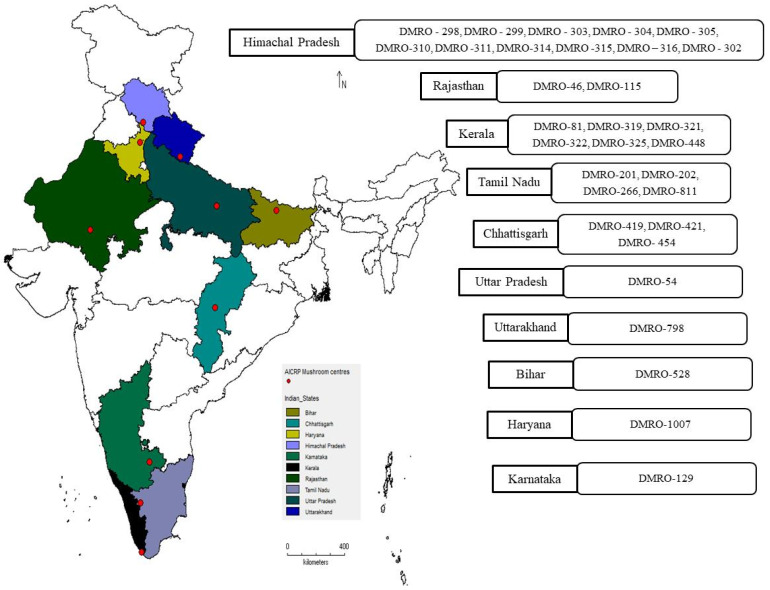
Geo-referencing map represents the different states of India. All thirty-three wild strains of *C. indica* were deposited by these states in the culture bank of ICAR-DMR, Solan, Himachal Pradesh, India.

Thirty-three strains of *C. indica* were procured from the culture bank, ICAR-Directorate of Mushroom Research (ICAR-DMR), Solan, Himachal Pradesh, India. These strains were collected and deposited in the culture bank of ICAR-DMR by different research organizations affiliated with the All India Coordinated Research Project (AICRP) mushroom network spread over different states of India *viz.* Karnataka, Rajasthan, Uttar Pradesh, Kerala, Tamil Nadu, Haryana, Chhattisgarh, Bihar, Himachal Pradesh, and Uttarakhand (Figure 1). To maintain the cultures, malt extract (20 g) and agar (20 g) (HiMedia, Mumbai, India) were mixed in one litre distilled water (pH-7.0) followed by autoclaving (121 °C, 30 min.), sub-culturing and incubation at 32 ± 2 °C at ICAR-DMR culture bank [28]. A temperature of 16 ± 1 °C was maintained for long-term storage of the cultures. 

### 2.2. Molecular Identification of the Different Strains of C. indica

DNA of the thirty-three strains of *C. indica* was isolated and subjected to internal transcribed spacer (ITS)-based PCR amplification followed by sequencing for the molecular identification. The PCR was performed using universal ITS1 (5′-TCCGTAGGTGAACCTGCGG-3′) and ITS4 (5′-TCCTCCGCTTATTGATATGC-3′) primer specific to rDNA region [29]. The following PCR conditions were used for the amplification: initial denaturation at 94 °C for 5 min followed by 35 cycles of denaturation (94 °C for 30 s), annealing (57 °C for 30 s) and extension step (72 °C for 90 s) along with a final extension at 72 °C for 7 min. Further, these samples were subjected to sequencing (Eurofin, Bengaluru, India) followed by trimming of the raw/low quality sequences using the DNA-based sequence assembly software (DNABaser, Romania). Further, the sequencing results of ITS1 and ITS4 were aligned to create a consensus using the MEGA X tool [30]. This consensus sequence was used as a query to search in the nucleotide BLAST-NCBI (blast.ncbi.nlm.nih.gov) analysis. 

### 2.3. Agronomical Evaluation

The spawn for all the thirty-three strains of *C. indica* was prepared as per the procedure described by Sharma and Kumar [31]. To assess the agronomic performance of the strains, a cultivation trial was conducted under controlled environmental conditions. The freshly procured wheat straw substrate was used for the cultivation and prepared using the steam (tunnel) pasteurization method as described by Nath et al. [32]. Layer spawning (5% on wet weight basis) was performed in polyethylene (PE) bags. Spawned substrate (5 kg) was filled in the PE bags and then shifted to the cropping rooms of ICAR-DMR, Solan, Himachal Pradesh, India. Casing (75% garden soil and 25% sand mixture) was applied to the substrate in the PE bags after completion of spawn run. Tunnel pasteurized-garden soil and sand mixture (75:25) were used as casing material. The temperature of 32 ± 2 °C was maintained during the cultivation including spawn run and fruiting. Illumination (1000 lux; 6 h) and relative humidity (85 ± 5%) were maintained during the formation of fruit-bodies. The experiment was conducted in randomized block design in the cropping room and randomization was performed in racks, both at spatial and longitudinal levels.

### 2.4. Morphological Characterization

Different morphological parameters *viz.* average fruit body weight (g), pileus length (cm), and stipe length/width (cm) were recorded for all the strains along with commercially released strain, i.e., the control (DMRO-302). The biological efficiency (BE)/yield was also calculated for all the strains as per the formula described by Nath et al. [32]. Further, to calculate the percentage increase in BE of particular strain over the control (DMRO-302), the following formula was used: BE (%) of (test strain − control strain/test strain) × 100. Only those strains were selected as high yielding strains which showed minimum ten percent higher yield as compared with the control strain.

### 2.5. Genetic Diversity Analysis Using SRAP Markers

The SRAP molecular marker system was used to analyze the diversity of the thirty-three strains of *C. indica.* The ten SRAP primers/combinations (Supplementary Table S1) were used for the PCR profiling of the thirty-three strains of the milky mushroom along with the control strain. The following PCR conditions were utilized for amplification: Initial denaturation at 94 °C for 3 min; and five cycles of denaturation: 94 °C for 60 s; annealing: 35 °C for 90 s; extension: 72 °C for 90 s. Further, thirty-five cycles of denaturation (94 °C for 60 s); annealing (50 °C for 90 s) and extension (72 °C for 90 s) along with final extension (72 °C for 8 min) were followed for the completion of the PCR amplification. The PCR product was analyzed using Agarose (1.5% Hi-Media, Mumbai, India) gel electrophoresis in Tris-borate-EDTA (TBE; HiMedia, Mumbai, India) buffer followed by ethidium bromide staining and visualization using Alpha Imager Gel Doc (Alpha Innotec, San Leandro, CA, USA). The DNA ladder L_1_ (1 kb DNA Ladder, Takara Bio. Inc., New Delhi, India) and L_2_ (100 bp DNA Ladder, Takara Bio. Inc., New Delhi, India) were used to determine the amplicon size.

### 2.6. Proximate Analysis of High Yielding Strains of C. indica

The proximate analysis was performed for the eight high yielding strains (DMRO-299, DMRO-321, DMRO-202, DMRO-298, DMRO-54, DMRO-81, DMRO-454 and DMRO-303) along with the control (DMRO-302). Crude fat/fiber, ash content, protein, carbohydrate, β-carotene, lycopene, DPPH activity, and phenolic content were estimated for these strains. All the experiments were conducted in triplicate with completely randomized design (CRD). The methodology for the proximate analysis was adopted from Sharma et al. [33].

### 2.7. Statistical and Software Analysis

The significance of each parameter was tested via the F-test at a 5% significance level using OPSTAT [34]. The morphological parameters were analyzed for principal component analysis (PCA). The objective of PCA was to discover relationships between independent variables using XLSTAT software [35]. The relationship between the groups formed in the PCA and mean data of phenotypic characters *viz.* days of first harvest, average fruit body weight, pileus length, stipe length/width and biological efficiency (%) was established. Moreover, the PCR amplicons generated through the SRAP markers/combinations were scored as present (1) and absent (0). Further, scoring binary data was utilized for clustering analysis via the SIMQUAL/SAHN module and Jaccard similarity coefficient. Further, Unpaired Group Mean Algorithm (UPGMA) was used to generate the dendrogram (tree) using the NTSYSpc 2.0i software [36]. The PIC of marker was determined via the POWERMARKER software (version-3.25). 

## 3. Results and Discussion

### 3.1. Molecular Identification and Morphological Yield Analysis of the Thirty-Three Strains of C. indica

**Figure 2:**
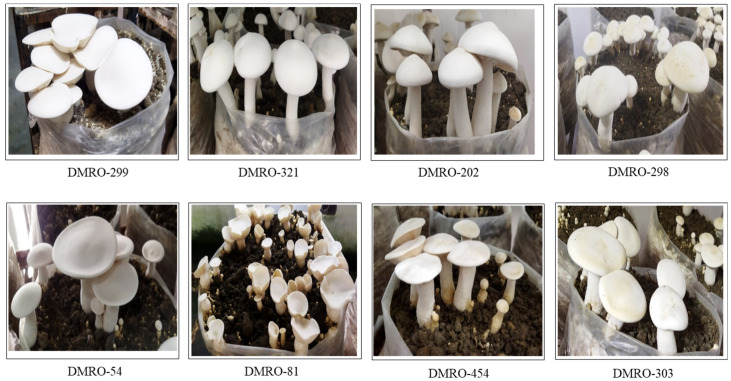
Morphological features of fruit body in eight high yielding strains of *C. indica*.

Internal transcribed spacer (ITS) of nuclear DNA is a well-established molecular identification/barcoding tool for different fungal strains [37]. Therefore, ITS-based PCR amplification and sequencing of the thirty-three strains were performed for the molecular identification. The results of NCBI- BLAST analysis of these strains confirmed their identity as *C. indica.* All these sequences were submitted to the NCBI database (Supplementary Table S2). These thirty-three strains of *C. indica* were subjected to cultivation trial along with the control, i.e., commercially released strain DMR Milky-334 (DMRO-302) [10,11]. Further, the morphological and yield-related parameters were recorded along with the control (DMRO-302). A significant effect was observed in all the strains through analysis of variance study (Supplementary Table S3). Maximum (48.33 days) and minimum (37.00 to 37.33 days) number of days of the first harvest were recorded in two strains (DMRO-314 and DMRO-315) and nine strains, respectively. Average fruit body weight was maximum in DMRO-202 (92.33 g) followed by DMRO-54 (67.33 g), while it was minimum in DMRO-325 (7.67 g). Variations were observed for pileus (cap) length in the thirty-three strains, ranging from 4.00 to 10.17 cm. While maximum pileus (cap) length was observed in DMRO-202 (10.17 cm) followed by DMRO-299 (9.60 cm), minimum pileus length was recorded in DMRO-325 (4.03 cm). On the other hand, highest stipe length was recorded in DMRO-299 (14.80 cm) followed by DMRO-46 (9.30 cm). Minimum stipe length was observed in DMRO-81 (2.17 cm). Stipe width diameter was maximum in DMRO-454 and DMRO-528 (2.97 cm) and minimum in DMRO-325 (1.13 cm). Notably, highest yield (BE %) was observed in DMRO-299 (64.08%) followed by DMRO-321(56.84%). The minimum yield was recorded in DMRO-304 (28.70%). Overall, evaluation of the yield (BE%) analysis of the thirty-three strains of *C. indica* identified eight high yielding strains as compared with the control (DMRO-302) (Figure 2).

In a similar study, cultivation trial analysis of the twenty-five strains of *C. indica* established CBE-TNAU-1523 strain as a high yielding strain (194.5% BE) followed by APK-2 (184.5% BE) [14]. Similarly, Kavitha et al. [38] reported maximum yield in Bheema (157% BE) as compared with IIHR Ca-1 (104% BE) and APK-2 (101% BE). Moreover, APK-2 of *C. indica* was reported as good yielding strain with short cropping cycle [8]. Another similar study evaluating five *C. indica* strains reported maximum yield in CI-6 (111.9% BE) [12] and CI-14 (81.16% BE) [13], while Singh et al. [15] analyzed the yield potential of seven strains and reported one (Ci-06) as a high yielding strain (57.3% BE). Similarly, one high yielding strain (CI-524; 69.66% BE) was reported from Chhattisgarh, India, after evaluation of the five strains of *C. indica* [39]. In general, few strains of the same geographical region were analyzed for their yield potential in various studies. The present study explored the different wild strains of various geographical regions of India and identified eight high yielding strains in *C. indica* as compared with the commercially released strain (DMRO-302). In this study, we have also observed morphological variations in the different strains *viz.* campanulate cap shape with incurved margin (DMRO-321, DMRO-202 and DMRO-316); brown pigmented cap/scurfy scales in stipe (DMRO-320, and DMRO-325) and convex to irregular cap shape (DMRO-528) (Supplementary Figure S1). Similarly, Bhupathi and Subbaiah [14] reported variations in color and fruit body (pileus-stipe) shape in different strains of *C. indica*.

### 3.2. Principal Component Analysis

**Figure 3:**
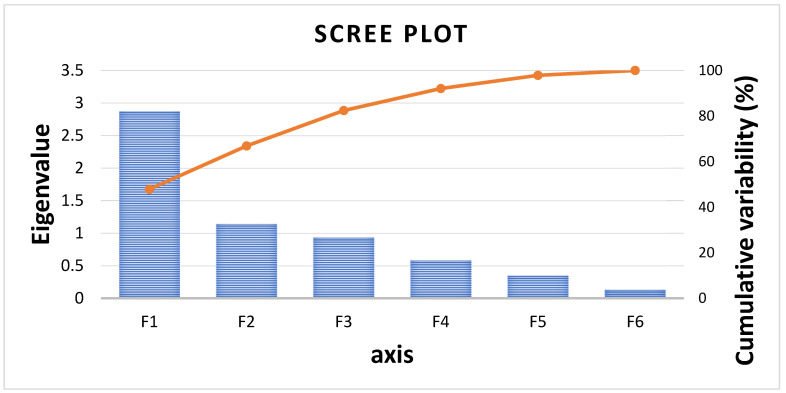
Scree plot demonstrates total variability captured by each principal component.

**Figure 4:**
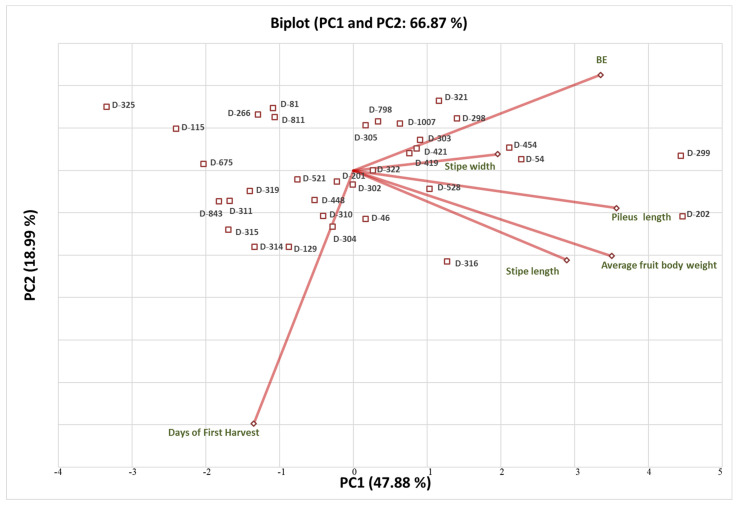
PCA biplot (PC1 and PC2) showing the relation of strains of *C. indica* with different morphological parameters. D: DMRO.

**Figure 5:**
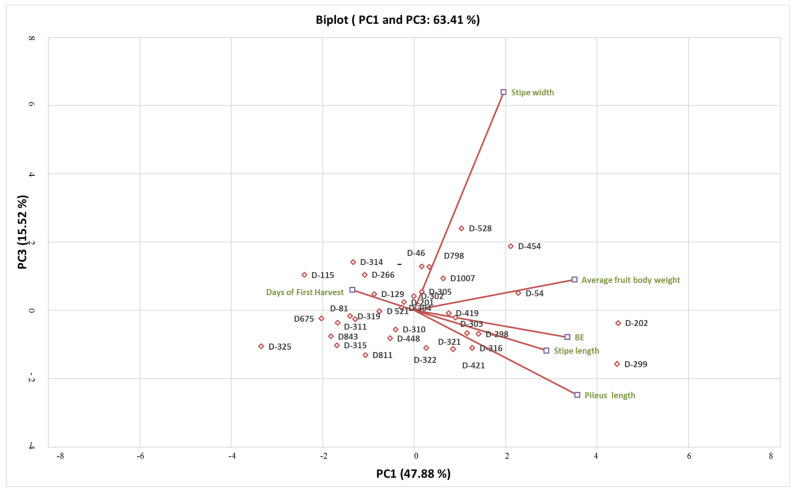
PCA biplot (PC1 and PC3) showing the relation of strains of *C. indica* with different morphological parameters. D: DMRO, BE: Biological Efficiency.

A total six components were found in the PCA analysis. The eigen values of the components were 2.873 (PC1), 1.139 (PC2), 0.931 (PC3), 0.581 (PC4), 0.346 (PC5) and 0.129 (PC6). The per cent variability of each component was 47.88 (PC1), 18.99 (PC2), 15.52 (PC3), 9.67 (PC4), 5.77 (PC5) and 2.15 (PC6). Three principal components, PC1, PC2 and PC3, were found relevant and important, accounting a total of 82.39% of variation. In the scree plot, X and Y axis shows principal components and eigen values, respectively (Figure 3). The PC1 component was directly correlated with BE, stipe width, pileus length, average fruit body weight and stipe length (Figure 4). The PC2 and PC3 correlated with days to harvest (Figure 4 and Figure 5). On the basis of PC1 and PC2 biplot, it can be seen that DMRO-298, DMRO-321, DMRO-303, DMRO-1007, DMRO-798, DMRO-305, DMRO-419, DMRO-421, and DMRO-322 strains formed one group for BE. The stipe width group contains DMRO-299, DMRO-454, DMRO-419, DMRO-421, and DMRO-54 strains. Moreover, the strains grouped under pileus length, average fruit body weight and stipe length were DMRO-202, DMRO-528, DMRO-322 and DMRO-316. The PCA biplot revealed that BE has a low angle between pileus length and average fruit body weight and were mostly correlated. Pileus length, average fruit body weight and stipe length have low angles which indicate positive correlation with each other (Figure 2). A similar analysis was also performed for selection in the different strains of *A. bisporous* [40].

### 3.3. Correlation and Cluster Analysis of the Thirty-Three Strains of C. indica

Pearson correlation analysis indicates significant positive correlation between BE and pileus length, stipe length, and average fruit body weight at significance level (*p* = 0.05). However, no significant correlation was observed between BE and stipe width. A negative significant correlation was found between days to first harvest and BE. The average fruit body weight was positively correlated with pileus length, and stipe length/width. Moreover, positive correlation was observed for pileus length and stipe length (Supplementary Table S4).

**Figure 6:**
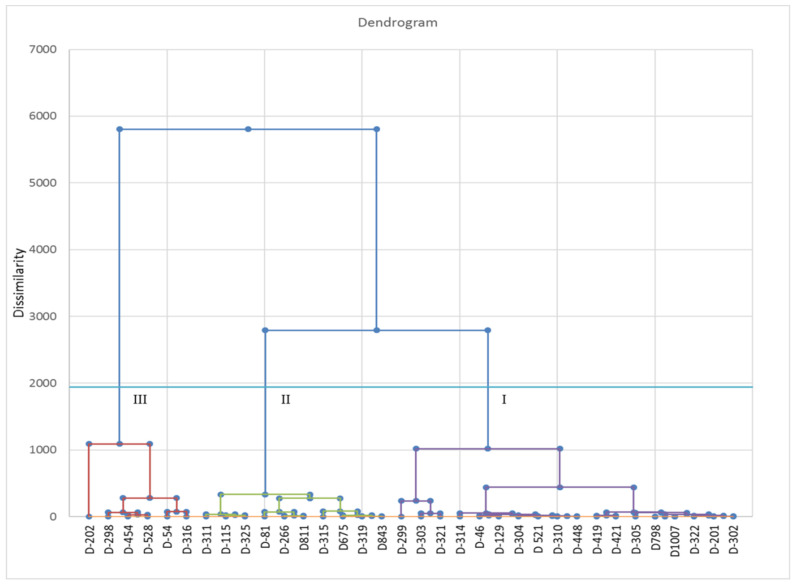
Euclidean distance and Ward method based morphological cluster analysis of the thirty-three strains along with control (DMRO-302). D: DMRO.

The maximum coefficient of variation was found for stipe length followed by average fruit body weight among morphological characters of the thirty-three strains. The most heritable character was days of first harvest followed by average fruit body weight. The genetic advance was maximum for average fruit body weight followed by stipe length. The highest genotypic and phenotypic coefficient of variation was observed for average fruit body weight and stipe length, respectively (Supplementary Table S5). These findings suggest selection preference for the average fruit body weight instead of stipe length which depends on the environmental parameters. Cluster analysis based on Euclidean distance (Ward method) constituted three clusters (Cluster I-III) (Figure 6). Cluster I contains maximum strains, i.e., eighteen strains (DMRO-46, DMRO-129, DMRO-201, DMRO-299, DMRO-303, DMRO-304, DMRO-305, DMRO-310, DMRO-314, DMRO-321, DMRO-322, DMRO-419, DMRO-421, DMRO-448, DMRO-521, DMRO-798, DMRO-1007 and DMRO-302). Cluster II and III have six and ten strains, respectively. Cluster I was observed as the largest cluster based on dissimilarity distance and distance to centroid was 26.30. Moreover, cluster variance was more for cluster II, followed by cluster I (Supplementary Table S6). Cluster I and III were more distant, thus hybridization between these can be utilized to achieve high variability. Notably, cluster I, II and III contain three, four and one high yielding strains, respectively. Overall, the morphological cluster analysis indicates the variations among different strains.

### 3.4. Phylogenetic Analysis of Different Strains of C. indica

**Figure 7:**
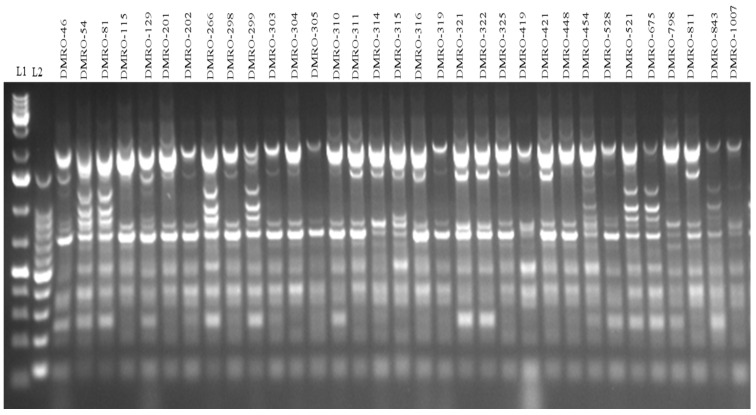
Representative SRAP-based PCR profiling (Me7 + em7 primer combination) in the thirty-three strains of *Calocybe indica* using primer. DNA ladder: L1 and L2.

**Figure 8:**
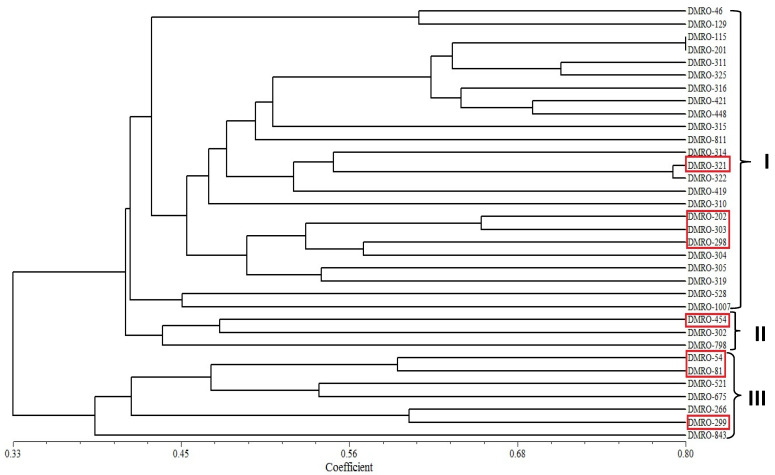
UPGMA-based cluster analysis of the thirty-three strains of *C. indica* along with control (DMRO-302). Red box reveals the eight high yielding strains.

SRAP marker system is an effective method for the analysis of genetic diversity of the different strains of mushroom. In this study, thirty-three strains of *C. indica* were analyzed using ten SRAP markers/combinations (Figure 7). SRAP-based genetic diversity analysis categorized these strains into three clusters. Maximum strains (24) were grouped into cluster I followed by cluster III (7 strains). Four high yielding strains (DMRO-321. DMRO-202, DMRO-303, and DMRO-298) were observed in cluster I followed by cluster III (DMRO-54, DMRO-81, and DMRO-299), while only one high yielding strain (DMRO-454) was grouped in cluster II. The highest yielding strain (DMRO-299) was grouped into cluster III (Figure 8), while the control strain (DMRO-302) was observed in cluster II. Ten SRAP primer pairs amplified a total of 211 alleles with an average of 21.1 alleles/primer pair, out of which 198 (93.84%) were polymorphic. The maximum alleles (32) were observed for me7/em4 primer combination while minimum (11) was observed in me7/em5 primer. The PIC value ranges from 0.200 to 0.265, and a mean of 0.232 was estimated for all the primer combinations, which indicates high variability among the different tested strains. The lowest PIC value (0.200) was recorded for the me9/em2 marker, closely followed by the me9/em5 marker (0.201). Whereas, the highest PIC value was obtained for the me7/em7 marker (0.265), closely followed by the me6/em5 marker (0.262).

Liu et al. [27] analyzed the genetic diversity of twenty-four widely cultivated strains of edible mushroom (*Cyclocybe chaxingu*) using SRAP markers/combinations and observed 79.52% polymorphic fragments. Moreover, Fu et al. [18] performed genetic diversity analysis of twenty-three strains of *L. edodes* and reported 56.3% polymorphic fragments. In this study, we observed a total of 211 loci, out of which 198 loci (93.84%) were polymorphic. The high level of polymorphism observed in this study corroborated the SRAP-based genetic diversity analysis of nineteen strains of *A. polytricha* (95.9%) [20]; and thirty-four strains of *A. auricularia* (96.1%) [21]. On the other hand, Du et al. [41] utilized the SRAP markers to investigate the genetic diversity of twenty wild and four cultivated strains of the *A. polytricha* and reported 99.8% and 40.7% polymorphism, respectively. Similarly, we have observed high levels of polymorphisms in this study, which further indicate the diversity among the different tested strains.

### 3.5. Nutritional Analysis of the Eight High Yielding Strains of C. indica

**Figure 9:**
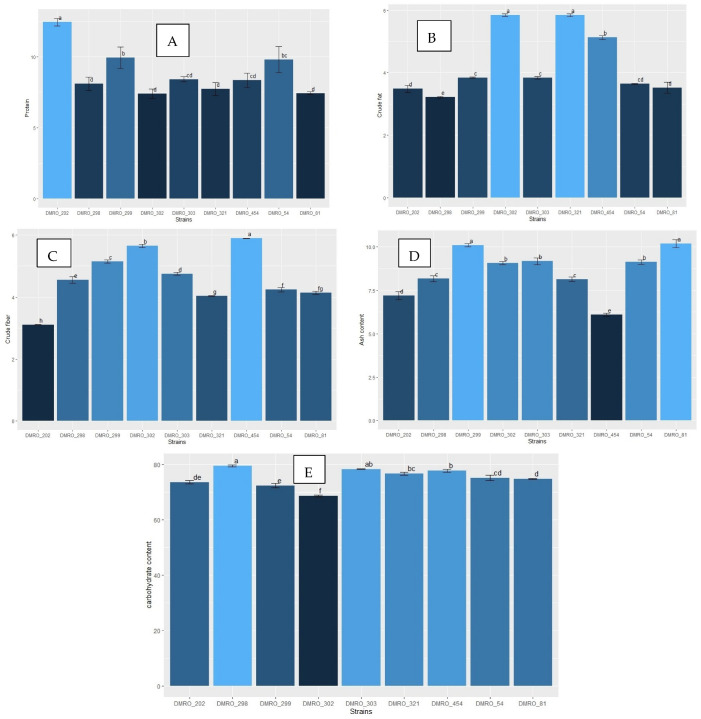
Nutritional profiling of the eight high yielding strains along with the control (DMRO-302). (**A**) Protein, (**B**) Crude fat, (**C**) Crude fiber, (**D**) Ash content and (**E**) Carbohydrate content.

**Figure 10:**
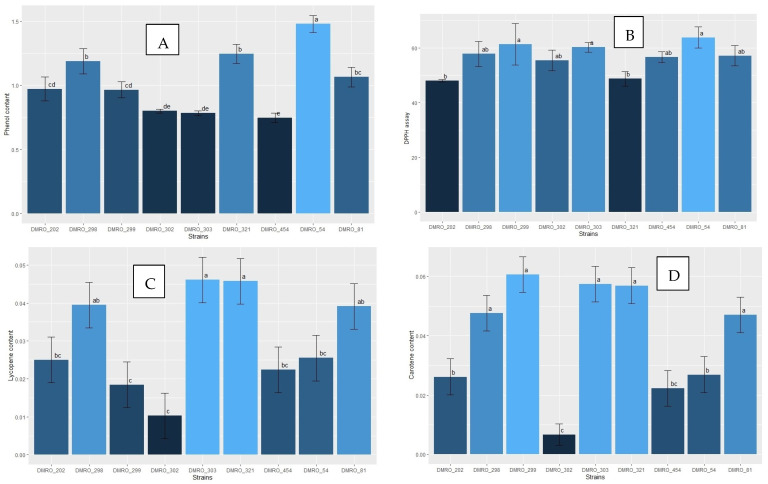
Bioactive compound analysis of the eight high yielding strains along with the control (DMRO-302). (**A**) Phenol content, (**B**) Antioxidant activity by DPPH assay, (**C**) Lycopene content, and (**D**) β-carotene content.

Mushrooms are well known for their high nutritive value and bioactive compounds. Significant variations were reported in nutritive composition among different mushroom species. Fruit body developmental stages, substrate composition and environmental factors also play an important role in nutritional composition variations among different mushrooms [42,43]. In the present study, proximate analysis was carried out for different nutritional parameters of high yielding strains along with a control (DMRO-302) (Figure 9 and Figure 10). All the proximate parameters were statistically significant. Highest crude fat content was observed in DMRO-321 and DMRO-302 (control) (5.84 to 5.85%) as compared with other strains. Lowest crude fat content was found in DMRO-298 (3.22%). Maximum crude fiber content was observed in DMRO-454 (5.90%), while minimum was recorded in DMRO-202 (3.10%). Significantly high ash content was observed in DMRO-81 and DMRO-299 (10.10 to 10.20%), while lowest ash content was recorded in DMRO-454 (6.1%). Protein content estimation revealed considerable variations among the tested strains, ranging from 7.40% to 12.44%. Significantly high protein content was recorded in DMRO-202 (12.44%) followed by DMRO-299 (9.93%). Maximum carbohydrate content was observed in DMRO-298 (79.52%) followed by DMRO-303 (78.28%). In all the strains, high carbohydrate content (72.33–79.52%) was observed as compared with the control (68.6%). Previous proximate studies reported variations in nutritional composition of different strains of *C. indica viz.* protein (17.69 to 27.25%); carbohydrate (49.06 to 64.26%); fats (3.13 to 4.96%); ash (7.43 to 12.80%) and fiber (3.40 to 14.07%) [43,44,45].

Dhakad et al. [46] evaluated five strains of *C. indica* and reported 2.37 to 13.42 μg/g phenol content. In our study, higher phenol content was recorded in DMRO-54 (1.48 mg/g), though similar phenol content was observed in DMRO-454, DMRO-303 and DMRO-302 strains (0.75 to 0.8 mg/g). Moreover, Viswakarma et al. [47] analyzed the bioactive compounds of the local strains of *C. indica* and reported 1.9 μg/mg β-carotene and 0.098 μg/mg lycopene content. In this study, high β-carotene content was observed in DMRO-299, DMRO-321 and DMRO-303 strains (0.06 μg/g). Moreover, DMRO-321 and DMRO-303 strains also possess higher lycopene content (0.05 μg/g) as compared with other high yielding strains. Additionally, DPPH radical scavenging (antioxidant) activity analysis revealed maximum activity in DMRO-54 (63.83%) and DMRO-299 (61.41%) strains as compared with other strains. However, the substrate types/supplements used for the cultivation of the mushroom were reported to influence the proximate composition and antioxidants [48].

## 4. Conclusions

*Calocybe indica* is one of the most popular mushroom in tropical and subtropical countries. The growing ability of this mushroom in high temperatures and longer shelf life increased its demand and adoption rate among the mushroom growers. Viewing the current and future demand, morphological and molecular evaluation of the thirty-three strains of *C. indica* was explored and the results showed variations among the tested strains. The findings of this study suggest diversity at morphological, molecular and proximate levels. In addition to this, average fruit body weight was observed as the most suitable trait for the selection of strains in breeding programs due to its medium heritability and comparatively high genetic advance. The identified eight high yielding strains will be helpful to promote commercialization as well as to enhance the income of mushroom growers. Overall, the findings of this study will accelerate future breeding programs of *C. indica.*

## Data Availability

Data is contained within the article.

## Supplementary Materials

The following supporting information can be downloaded at: https://www.mdpi.com/article/10.3390/foods12112119/s1, Figure S1: Morphological variations in different strains of *C. indica*. (A) Cap campanulate with incurved margin (DMRO-321, DMRO-202 and DMRO-316); (B) White to dull white cap with brownish pigmentation and stipe with scurfy scales (DMRO-320, and DMRO-325); and C. Convex to irregular cap shape (DMRO-528); Table S1: Ten SRAP primers/combinations used for genetic diversity analysis; Table S2: Thirty-three strains of *Calocybe indica* along with NCBI I.D.; Table S3: Mean, CD and SE of the characters studied for the thirty-three strains of *C. indica* along with the control (DMRO-302); Table S4: Correlation analysis; Table S5: Variability analysis; and Table S6: Cluster analysis.

## References

[1] Alam N., Amin R., Khan A., Ara I., Shim M.J., Lee M.W., Lee T.S. (2008). Nutritional analysis of cultivated mushrooms in Bangladesh—*Pleurotus ostreatus*, *Pleurotus sajor-caju*, *Pleurotus florida* and *Calocybe indica*. Mycobiology.

[2] Thakur M.P., Singh H. (2014). Advances in the cultivation technology of tropical mushrooms in India. JNKVV Res. J..

[3] Ghosh S., Acharya K. (2022). Milky mushroom: A healthy nutritious diet. Food Res. Int..

[4] Kamal S., Barh A., Sharma K., Sharma V.P., Kumar Srivastava D., Kumar Thakur A., Kumar P. (2021). Mushroom Biology and Advances. Agricultural Biotechnology: Latest Research and Trends.

[5] Chakraborty U., Sikdar S.R. (2010). Intergeneric protoplast fusion between *Calocybe indica* (milky mushroom) and *Pleurotus florida* aids in the qualitative and quantitative improvement of sporophore of the milky mushroom. World J. Microbiol. Biotechnol..

[6] Krishnamoorthy A.S., Balan V. (2015). A comprehensive review of tropical milky white mushroom (*Calocybe indica* P&C). Mycobiology.

[7] Shashikant M., Bains A., Chawla P., Fogarasi M., Fogarasi S. (2022). The current status, bioactivity, food, and pharmaceutical approaches of *Calocybe indica*: A review. Antioxidants.

[8] Krishnamoorthy A.S., Muthuswamy M.T., Nakkeeran S. (2000). Technique for commercial production of milky mushroom *Calocybe indica* P&C. Indian J. Mush..

[9] Rathore H., Sharma A., Prasad S., Kumar A., Shama S., Singh A. (2020). Yield, nutritional composition and antioxidant properties of *Calocybe indica* cultivated on wheat straw basal substrate supplemented with nitrogenous tree leaves. Waste Biomass Valorization.

[10] Sharma V.P., Kumar S., Sharma S. (2020). Technologies Developed by ICAR-DMR for Commercial Use.

[11] Sharma V.P., Heera G., Kumar S., Nath M. (2020). Development and identification of high yielding strain of *Calocybe indica* based on the multilocation trials. Mushroom Res..

[12] Sharma J.P., Kumar S. (2008). Evaluation of strains of milky mushroom (*Calocybe indica*) for cultivation in Jharkhand. Mushroom Res..

[13] Dhakad P.K., Chandra R., Yadav M.K., Patar U.R. (2015). Comparative study on growth parameters and yield potential of five strains of milky mushroom (*Calocybe indica*). J. Pure Appl. Microbiol..

[14] Bhupathi P., Subbaiah K.A. (2019). Comparison of colony morphology, sporophore characters and yield performance of wild and cultivated milky mushroom isolates. J. Pure Appl. Microbiol..

[15] Singh K., Sharma S., Kaur R., Sodhi H.S. (2020). Evaluation of *Calocybe indica* strains for lignocellulolytic enzymes and mushroom yield potential. Indian J. Hortic..

[16] Alzahib R.H., Migdadi H.M., Ghamdi A.A.A., Alwahibi M.S., Afzal M., Elharty E.H., Alghamdi S.S. (2021). Exploring genetic variability among and within Hail tomato landraces based on sequence-related amplified polymorphism markers. Diversity.

[17] Li G., Quiros C.F. (2001). Sequence-related amplified polymorphism (SRAP), a new marker system based on a simple PCR reaction: Its application to mapping and gene tagging in Brassica. Theor. Appl. Genet..

[18] Fu L.Z., Zhang H.Y., Wu X.Q., Li H.B., Wei H., Wu Q.Q., Wang L.A. (2010). Evaluation of genetic diversity in *Lentinula edodes* strains using RAPD, ISSR and SRAP markers. World J. Microbiol. Biotechnol..

[19] Liu J., Wang Z.R., Li C., Bian Y.B., Xiao Y. (2015). Evaluating genetic diversity and constructing core collections of Chinese *Lentinula edodes* cultivars using ISSR and SRAP markers. J. Basic Microbiol..

[20] Yu M., Ma B., Luo X., Zheng L., Xu X., Yang Z. (2008). Molecular diversity of *Auricularia polytricha* revealed by inter-simple sequence repeat and sequence-related amplified polymorphism markers. Curr. Microbiol..

[21] Tang L., Xiao Y., Li L., Guo Q., Bian Y. (2010). Analysis of genetic diversity among Chinese *Auricularia auricula* cultivars using combined ISSR and SRAP markers. Curr. Microbiol..

[22] Yao F., Lu L., Wang P., Fang M., Zhang Y., Chen Y., Zhang W., Kong X., Lu J., Honda Y. (2018). Development of a molecular marker for fruiting body pattern in *Auricularia auricula-judae*. Mycobiology.

[23] Du J., Guo H.-B., Li Q., Forsythe A., Chen X.-H., Yu X.-D. (2018). Genetic diversity of *Lepista nuda* (Agaricales, Basidiomycota) in Northeast China as indicated by SRAP and ISSR markers. PLoS ONE.

[24] Zhang Q.S., Xu B.L., Liu L.D., Yuan Q.Q., Dong H.X., Cheng X.H., Lin D.L. (2012). Analysis of genetic diversity among Chinese *Pleurotus citrinopileatus* Singer cultivars using two molecular marker systems (ISSRs and SRAPs) and morphological traits. World J. Microbiol. Biotechnol..

[25] Yin Y., Liu Y., Li H., Zhao S., Wang S., Liu Y., Wu D., Xu F. (2014). Genetic diversity of *Pleurotus pulmonarius* revealed by RAPD, ISSR, and SRAP fingerprinting. Curr. Microbiol..

[26] Barh A., Kamal S., Sharma V.P., Sharma K., Kumari B., Nath M. (2023). Identification and morpho-molecular characterization of low spore strain in oyster mush-room. Mol. Biol. Rep..

[27] Liu J., Ding F., Song H., Chen M., Hu D. (2022). Analysis of genetic diversity among Chinese *Cyclocybe chaxingu* strains using ISSR and SRAP markers. PeerJ.

[28] Kumar R., Singh G., Pandey P., Mishra P. (2011). Cultural, physiological characteristics and yield attributes of strains of milky mushroom (*Calocybe indica*). J. Mycol. Plant Pathol..

[29] White T.J., Bruns T.D., Lee S.S., Taylor J.W., Innis M.A., Gelf D.H., Sninsky J.J., White T.J. (1990). Amplification and direct sequencing of fungal ribosomal RNA genes for phylogenetics. PCR Protocols: A Guide to Methods and Application.

[30] Kumar S., Stecher G., Li M., Knyaz C., Tamura K. (2018). MEGA X: Molecular evolutionary genetics analysis across computing platforms. Mol. Biol. Evol..

[31] Sharma V., Kumar S., Singh M., Vijay B., Kamal S., Wakchaure G.C. (2011). Spawn production technology. Mushrooms: Cultivation, Marketing and Consumption.

[32] Nath M., Barh A., Sharma A., Bijla S., Bairwa R.K., Kamal S., Sharma V. (2022). Impact of the different casing material on the yield of *Calocybe indica* in polyethylene bag and bed system. Mushroom Res..

[33] Sharma V.P., Kumari B., Barh A., Kamal S., Kashyap R., Annepu S.K. (2021). Biochemical profiling and cultivation of medicinal fungus *Isaria cicadae* (Ascomycetes) from India. Int. J. Med. Mushrooms.

[34] Sheoran O.P., Tonk D.S., Kaushik L.S., Hasija R.C., Pannu R.S., Hooda D.S., Hasija R.C. (1998). Statistical Software Package for Agricultural Research Workers. Recent Advances in Information Theory, Statistics & Computer Applications.

[35] Vidal N.P., Manful C.F., Pham T.H., Stewart P., Keough D., Thomas R.H. (2020). The use of XLSTAT in conducting principal component analysis (PCA) when evaluating the relationships between sensory and quality attributes in grilled foods. MethodsX.

[36] Rohlf F.J. (1998). NTSYS-pc: Numerical Taxonomy and Multivariate Analysis System.

[37] Bellemain E., Carlsen T., Brochmann C., Coissac E., Taberlet P., Kauserud H. (2010). ITS as an environmental DNA barcode for fungi: An in silico approach reveals potential PCR biases. BMC Microbiol..

[38] Kavitha K., Latha R., Thirukumaran K. (2020). Assessment of Milky Mushroom Varieties in Kanyakumari District, India. Int. J. Curr. Microbiol. Appl. Sci..

[39] Kerketta A., Singh H.K., Shukla C.S. (2018). Cultivation of milky mushroom collected from different region of Chhattisgarh state. Int. J. Chem. Stud..

[40] Barh A., Sharma V.P., Annepu S.K., Kumari B., Kamal S., Shirur M., Sharma K. (2020). Selection of superior transgressive segregants from NBS-5 strain of white button mushroom. Mushroom Res..

[41] Du P., Cui B.K., Dai Y.C. (2011). Genetic diversity of wild *Auricularia polytricha* in Yunnan Province of South-western China revealed by sequence-related amplified polymorphism (SRAP) analysis. J. Med. Plants Res..

[42] Pedneault K., Angers P., Avis T.J., Gosselin A., Tweddell R.J. (2007). Fatty acid profiles of polar and non-polar lipids of *Pleurotus ostreatus* and *P. cornucopiae* var. ‘*citrino-pileatus*’ grown at different temperatures. Mycol. Res..

[43] Sumathy R., Kumuthakalavalli R., Krishnamoorthy A.S. (2015). Proximate, vitamin, aminoacid and mineral composition of milky mushroom, *Calocybe Indica* (P&C). Var. Apk2 commonly cultivated in Tamil nadu. J. Nat. Prod. Plant Resour..

[44] Pushpa H., Purushothoma K.B. (2010). Nutritional analysis of wild and cultivated edible medicinal mushrooms. World J. Dairy Food Sci..

[45] Manikandan K., Singh M., Vijay B., Kamal S., Wakchaure G.C. (2011). Nutritional and medicinal values of mushrooms. Mushrooms: Cultivation, Marketing and Consumption.

[46] Dhakad P.K., Chandra R., Yadav M.K., Patar U.R. (2017). Comparative Study on Nutraceuticals of Five Strains of Milky Mushroom (*Calocybe indica*). Int. J. Curr. Microbiol. Appl. Sci..

[47] Vishwakarma P., Singh P., Tripathi N.N. (2016). Nutritional and antioxidant properties of wild edible macrofungi from North-Eastern Uttar Pradesh, India. Indian J. Tradit. Knowl..

[48] Saranya V., Madhanraj P., Panneerselvam A. (2011). Cultivation, composting, biochemical and molecular characterization of *Calocybe indica* (C and A). Asian J. Pharm Res..

