# Supplementary material for: Identification of Eight High Yielding Strains via Morpho-Molecular Characterization of Thirty-Three Wild Strains of Calocybe indica
Source: Foods. 2023 May 24;12(11):2119. doi: 10.3390/foods12112119 (PMC10253166; doi:10.3390/foods12112119)
Supplement: Supplementary file 1 [file foods-12-02119-s001.zip › foods-2393466-supplementary/Supplementary Files/Supplementary Figures.pdf]

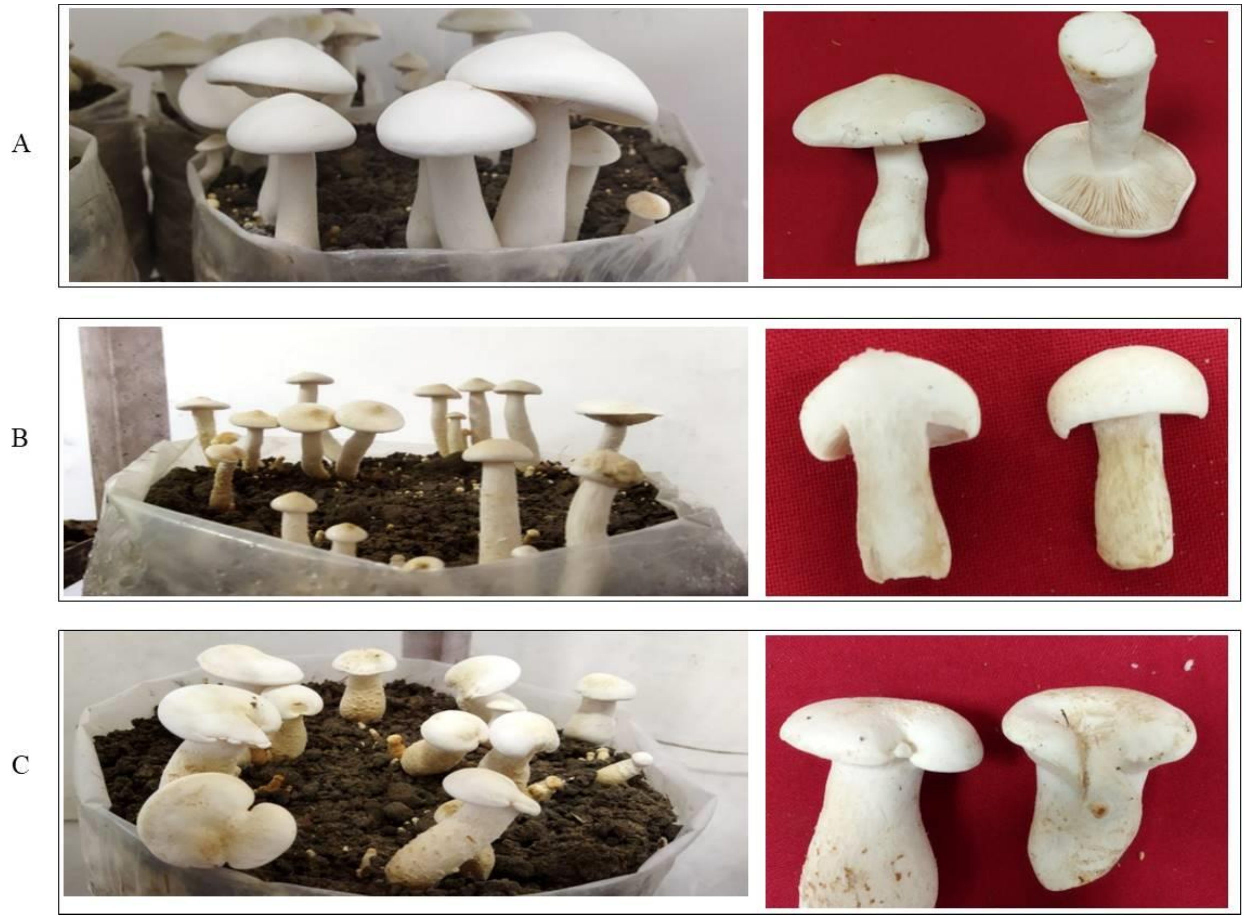

Figure S1. Morphological variations in different strains of *C. indica*. **A.** Cap campanulate with incurved margin (DMRO-321, DMRO-202 and DMRO-316); **B.** White to dull white Cap with brownish pigmentation and stipe with scurfy scales (DMRO-320, DMRO-325); and **C.** Convex to irregular cap shape (DMRO-528)
