# Supplementary material for: Identification of Eight High Yielding Strains via Morpho-Molecular Characterization of Thirty-Three Wild Strains of Calocybe indica
Source: Foods. 2023 May 24;12(11):2119. doi: 10.3390/foods12112119 (PMC10253166; doi:10.3390/foods12112119)
Supplement: Supplementary file 1 [file foods-12-02119-s001.zip › foods-2393466-supplementary/Supplementary Files/Supplementary Tables.pdf]

Table S1. Ten SRAP primers/combinations used for genetic diversity analysis

S. No.	SRAP marker/combination
1	ME7+em2
2	Me9+em2
3	Me8+em8
4	Me8+em5
5	Me7+em7
6	Me7+em4
7	Me6+em5
8	Me6+em9
9	Me9+em5
10	Me7+em5

Table S2. Thirty three strains of *Calcoybe indica* along with NCBI I.D.

S. No.	Strain	NCBI ID
1	DMRO-129	ON307076
2	DMRO-298	ON307077
3	DMRO-315	ON307078
4	DMRO-419	ON307079
5	DMRO-46	ON310887
6	DMRO-54	ON310888
7	DMRO-81	ON310889
8	DMRO-201	ON310890
9	DMRO-202	ON310891
10	DMRO-299	ON310892
11	DMRO-303	ON310893
12	DMRO-305	ON332080
13	DMRO-310	ON332081
14	DMRO-311	ON332082
15	DMRO-322	ON332083
16	DMRO-325	ON332084
17	DMRO-421	ON332085
18	DMRO-448	ON332086
19	DMRO-454	ON332087
20	DMRO-528	ON332088
21	DMRO-115	ON350811
22	DMRO-314	ON350812
23	DMRO-316	ON350813
24	DMRO-321	ON350814
25	DMRO-266	OP526972
26	DMRO-319	OP526973
27	DMRO-304	OP526974
28	DMRO-521	OP875091
29	DMRO-675	OP875092
30	DMRO-798	OP875093
31	DMRO-811	OP875094
32	DMRO-843	OP875095
33	DMRO-1007	OP875096

Table S3. Mean, CD and SE of the characters studied for thirty three strains of *C. indica* along with control (DMRO-302)

**Characters**	**Days to first harvest ***	**Cap length***	**Stipe length***	**Stipe width***	**Average fruit body weight***	**BE***
Mean	41.18	6.93	5.37	1.99	35.85	40.24
C.D.	1.16	2.28	5.01	0.69	20.73	12.22
SE(m)	0.41	0.81	1.77	0.24	7.32	4.32
SE(d)	0.58	1.14	2.50	0.35	10.36	6.11

* Based on one factor anova (P≤0.05) for all the characters

Table S4. Correlation analysis

Variables	Days of First Harvest	Pileus length	Stipe length	Stipe width	Average fruit boody weight	BE
Days of First Harvest	**1**	-0.197	-0.006	-0.136	-0.017	**-0.444**
Pileus length		**1**	**0.536**	0.109	**0.763**	**0.591**
Stipe length			**1**	0.210	**0.460**	**0.442**
Stipe width			0.210	**1**	**0.432**	0.291
Average fruit body weight					**1**	**0.490**
BE						**1**

Table S5. Variability analysis

	Days of First Harvest	Pileus length)	Stipe length	Stipe width	Average fruit body weight	BE
CV(%)	1.72	20.14	57.06	21.23	35.39	18.59
PCV(%)	8.58	25.10	64.83	27.46	56.21	26.67
GCV(%)	8.40	14.99	30.76	17.42	43.67	19.12
h2(%)	95.99	35.64	22.52	40.23	60.36	51.40
GAM(%)	16.96	18.43	30.07	22.76	69.90	28.24

Table S6. Cluster analysis

Cluster	I	II	III
Number of objects by cluster	18	6	10
Sum of weights	18	6	10
Within-cluster variance	123.273	307.400	95.325
Minimum distance to centroid	2.303	5.227	4.589
Average distance to centroid	9.230	13.740	8.791
Maximum distance to centroid	26.30	30.14	14.82
	D-46	D-54	D-81
	D-129	D-202	D-115
	D-201	D-298	D-266
	D-299	D-316	D-311
	D-303	D-454	D-315
	D-304	D-528	D-319
	D-305		D-325
	D-310		D-675
	D-314		D-811
	D-321		D-843
	D-322		
	D-419		
	D-421		
	D-448		
	D-521		
	D-798		
	D-1007		
	D-302		
